# Biplanar Reconstruction With Pectoralis Minor Tendon and Coracoacromial Ligament Transfer for Chronic Acromioclavicular Joint Dislocations

**DOI:** 10.1016/j.eats.2024.103104

**Published:** 2024-07-11

**Authors:** Marco Cartaya Méndez, Felipe Turner Ruiz-Tagle, Jorge Vargas Zúñiga

**Affiliations:** a Clínica las Condes, Santiago, Chile; b Hospital del Trabajador, Santiago, Chile

## Abstract

Acromioclavicular joint dislocation is a common pathology, affecting mostly young male patients. High-grade injuries require surgical treatment, but evidence is lacking regarding a gold standard technique. Chronic cases frequently are treated with graft reconstruction, but complications and availability remain as a limitation for autograft and allograft use, respectively. The objective of this Technical Note is to describe the treatment of chronic acromioclavicular dislocations by a reconstruction made with a local tendon graft, the pectoralis minor, in addition to transferring the coracoacromial ligament as a horizontal stabilizer.

The acromioclavicular (AC) joint represents the link between the clavicle and the scapula, which is responsible for the synchronized dynamic of the shoulder girdle.^1^ AC dislocations, with an incidence of 3 to 4 per 100,000 habitants, are a common occurrence. Most injuries occur during contact sports, affecting mostly young male patients.^2^ Symptomatic high-grade injuries (Rockwood IV-VI) require surgical management to restore AC joint stability. Patients presenting with injuries exceeding 2 weeks after occurrence are considered as having chronic lesions, leading to diminished clinical outcomes.^3^

Current evidence supports the use of biological augmentation in the surgical treatment of chronic AC dislocations.^4^ The semitendinosus tendon graft has become widely used in these cases. Despite satisfactory results published,^5^ its use as an autograft is associated with a high incidence of complications.^6^ The allograft option, meanwhile, is expensive and less available.

The primary objective of this Technical Note (Video 1) is to describe the treatment of the chronic AC joint dislocations by a reconstruction made with a local tendon graft, the pectoralis minor (Pm), as a vertical stabilizer plus the transfer of the coracoacromial ligament (CAL) as a horizontal stabilizer, in a patient with a Rockwood V AC dislocation of the shoulder (Fig 1). Advantages and disadvantages are shown in Table 1. Pearls and pitfalls are shown in Table 2.

**Fig 1:**
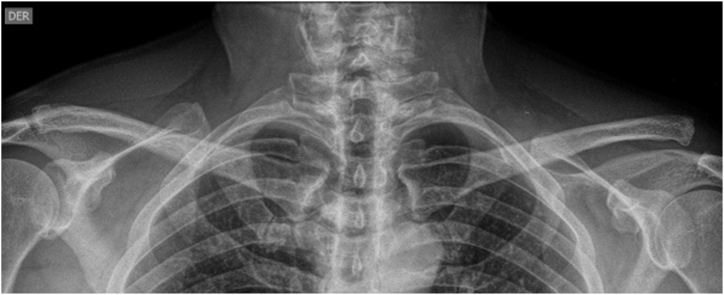
Preoperative anteroposterior acromioclavicular joint view showing a Rockwood V acromioclavicular joint dislocation of the left shoulder in an active male patient.

**Table 1:** Advantages and Disadvantages

Advantages	Disadvantages
One point required for graft healing Use of local autograft	Coracoacromial arch disruption Currently performed as an open technique
One single surgical approach	Cannot be used if coracoid fracture is present
Preserves CAL acromial insertion, maintaining its AC stabilizer function Theoretical effect of Pm tendon tenotomy on scapular dyskinesis	

AC, acromioclavicular joint; CAL, coracoacromial ligament; Pm, pectoralis minor.

**Table 2:** Pearls and Pitfalls

Pearls
Identification of the medial coracoclavicular ligaments is crucial to find the posterior border of the Pm tendon.
Coracohumeral ligaments must be completely released to achieve adequate CAL harvest and mobilization. If the Pm tendon graft is more than 4 mm in diameter, the inferior clavicular tunnel entrance can be widened by a FlipCutter to the desired width.
Pitfalls
Reduction and stability must always be achieved by the suspension systems before graft fixation. When acromioclavicular reduction is not possible, distal clavicular resection is indicated.

CAL, coracoacromial ligament; Pm, pectoralis minor.

## Surgical Technique

### Anesthesia and Patient Position

We use general anesthesia plus an interscalene block. A 45° beach-chair position is used.

### Surgical Approach

An anterosuperior oblique approach is used, located just medial to the AC joint. After the skin incision, the dissection continues through the subcutaneous tissue until the deltoid muscle is reached. A split is made above the coracoid.

### Graft Harvest and Preparation

**Fig 2:**
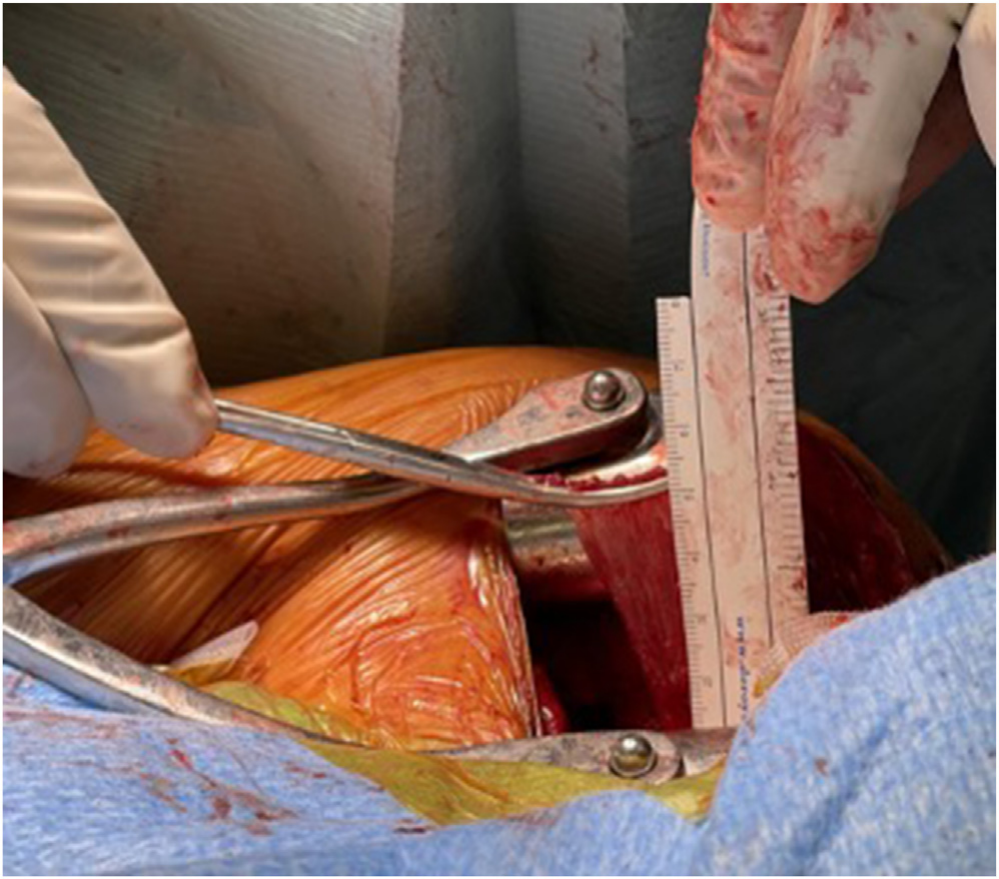
Once the musculotendinous junction is identified, the muscles fibers of the Pm tendon are detached using a picking technique. Any remaining fibers are then stripped off. The image shows the left shoulder of a patient in a beach-chair position. (Pm, pectoralis minor.)

For the Pm tendon harvest, medial coracoclavicular (CC) ligaments first must be identified. With a curved blunt dissector, a swab thread is passed from posterior to anterior between the medial CC ligament and the Pm tendon insertion at the coracoid. The thread is recovered from anterior between the Pm tendon and the conjoined tendon, making a loop around the Pm tendon. The Pm tendon is pulled up by the thread until the musculotendinous junction is identified under traction. With a picking technique (Video 1), the muscle fibers of the Pm are peeled off at this point, releasing the Pm tendon from the muscle belly while keeping its coracoid insertion. The remaining muscle fibers attached to the tendon are stripped off (Fig 2). The tendon is tubularized with a No. 2 FiberLoop suture (Arthrex, Naples, FL).

**Fig 3:**
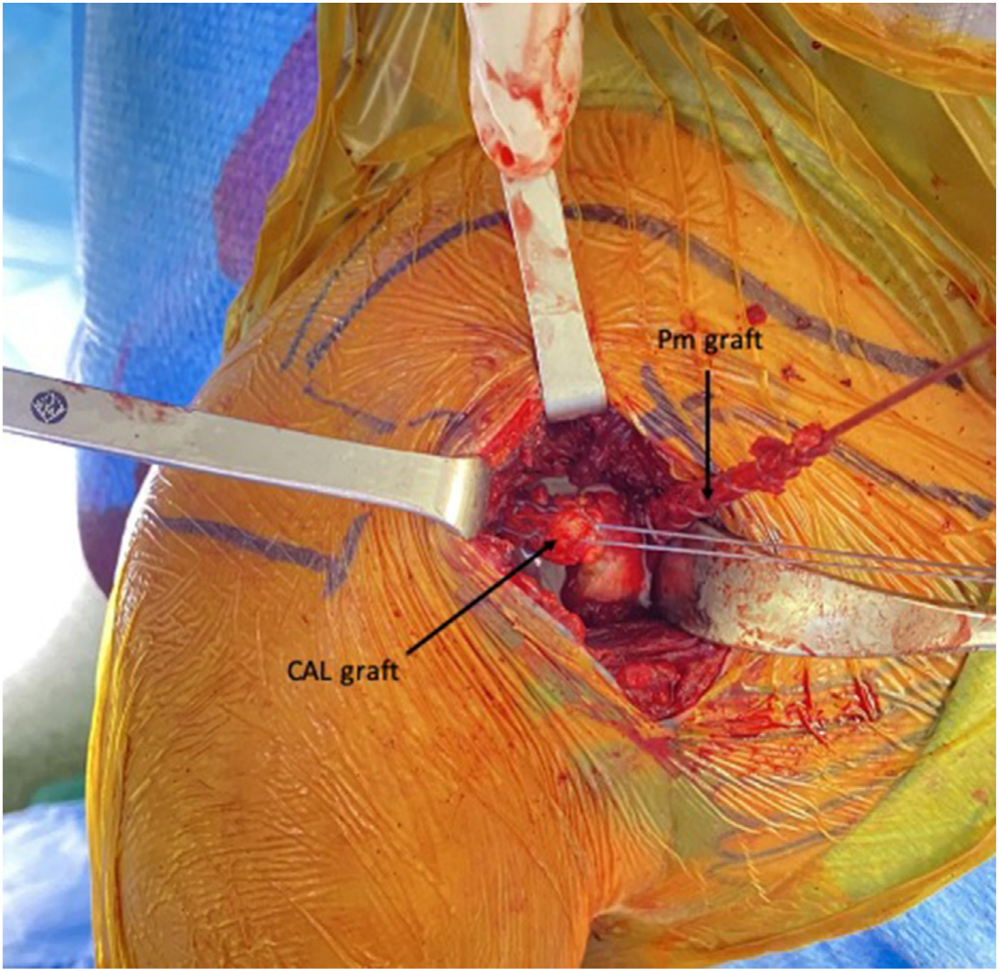
The CAL is identified, released from its coracoid insertion, and then prepared using a No. 2 FiberWire suture. The Pm tendon is tubularized with a No. 2 FiberLoop suture. The image shows the left shoulder of a patient in a beach-chair position. (CAL, coracoacromial ligament; Pm, pectoralis minor.)

After the Pm tendon harvest, the CAL is identified, released from its coracoid insertion using a No. 15 blade, and prepared with Krackow stitches using a No. 2 FiberWire (Fig 3). Two bands of the CAL have been found in some patients, in which case the anterior one is selected to be prepared.

### Implants and Graft Positioning

**Fig 4:**
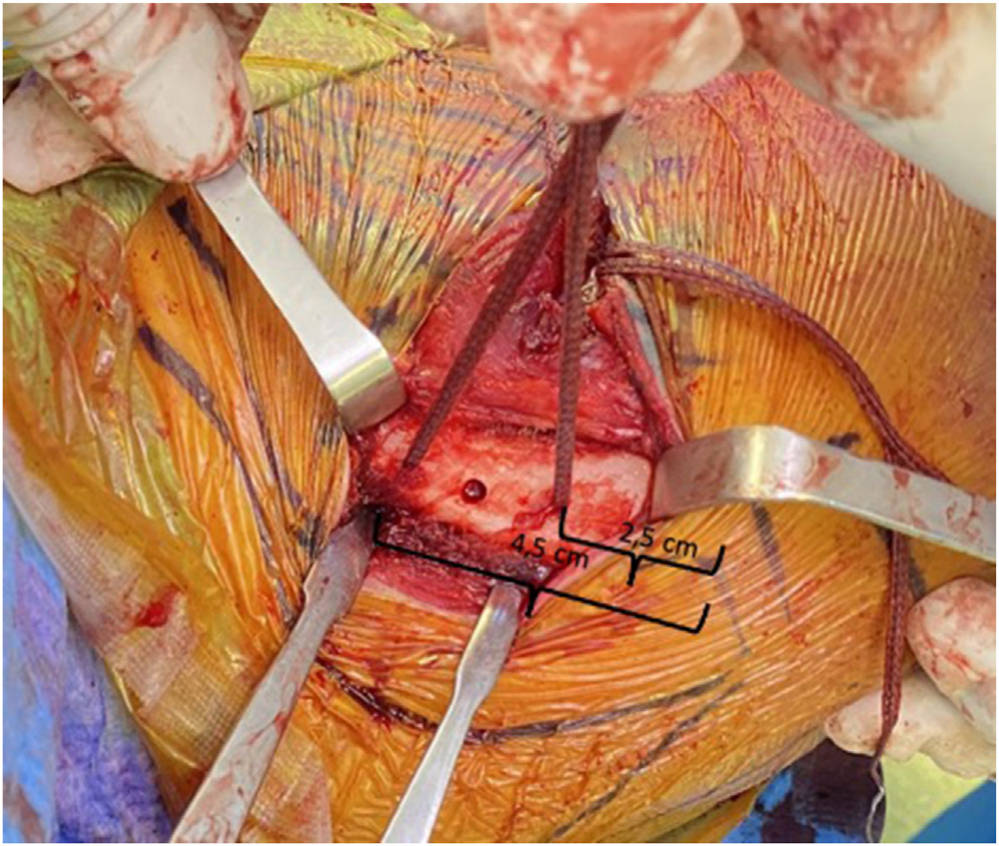
Three clavicular tunnels are drilled, with two 2.5-mm tunnels being drilled at 2.5 and 4.5 cm from the lateral border of the clavicle (as shown in the image with FiberTape sutures). The third tunnel, measuring 4.0 mm (indicated by the empty hole in the image), is drilled between them to allow for the fixation of the Pm tendon. The image shows the left shoulder of a patient in a beach-chair position. (Pm, pectoralis minor.)

A small Homman retractor is placed on the posterior surface of the clavicle to expose its superior surface. Two tunnels, each measuring 2.5 mm, are drilled at 2.5 and 4.5 cm from the lateral border of the clavicle. A FiberTape loop is then advanced through the medial tunnel, retrieved below the medial border of the coracoid base, and passed back through the lateral tunnel. A second FiberTape loop is advanced from the posterior border of the clavicle, retrieved below the coracoid from medial to lateral, and then grabbed from the anterior border of the clavicle, making a loop around the clavicle and under the coracoid. A third clavicular tunnel (4.0 mm) is drilled between the previous 2 tunnels, 3.5 cm from the lateral border of the clavicle (Fig 4).

The leading sutures of the Pm graft are then mobilized through the 4.0-mm clavicular tunnel in a retrograde manner by pulling them from above. If the graft is too wide, it may not pass through the tunnel, which would result in inadequate tension. In such cases, the tunnel needs to be gradually expanded until a tight fit is achieved.

### Reduction of the AC Joint

Once the grafts are prepared and the clavicular tunnels drilled, the AC joint reduction is accomplished by axial compression, pushing the clavicle downward with a graft impactor while manually pushing the humerus upward from the elbow. The reduction is checked under fluoroscopy. If the initial maneuver fails, the AC joint has to be explored to remove any interposed tissue.

### Implants and Grafts Fixation

**Fig 5:**
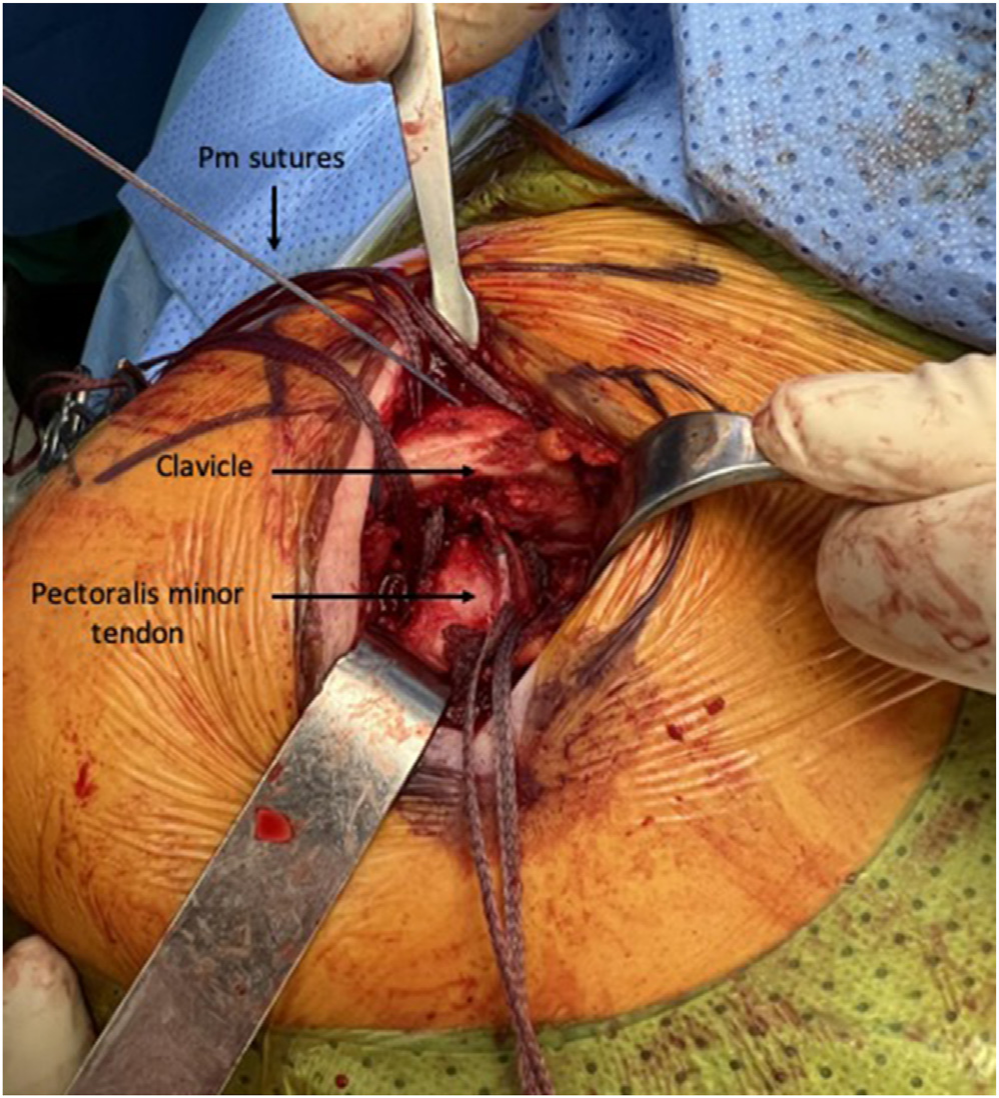
After the harvesting and tunnel drilling, the Pm leading sutures are mobilized retrogradely through the 4.0-mm clavicular tunnel by pulling them from the superior end. The image shows the left shoulder of a patient in a beach-chair position. (Pm, pectoralis minor.)

Once the AC reduction is achieved, the FiberTape loops are secured by Nice knots in the superior surface of the clavicle. Then the Pm tendon sutures are tightened through the 4.0-mm clavicular tunnel (Fig 5) and secured in the superior surface of the clavicle over a cortical button (ABS; Arthrex, Naples, FL). The anterolateral clavicle cortex is prepared with a rasp. Finally, the CAL sutures are passed under the deltoid fibers and secured by a 2.9-mm knotless suture anchor (PushLock; Arthrex) in the posterior cortex.

**Fig 6:**
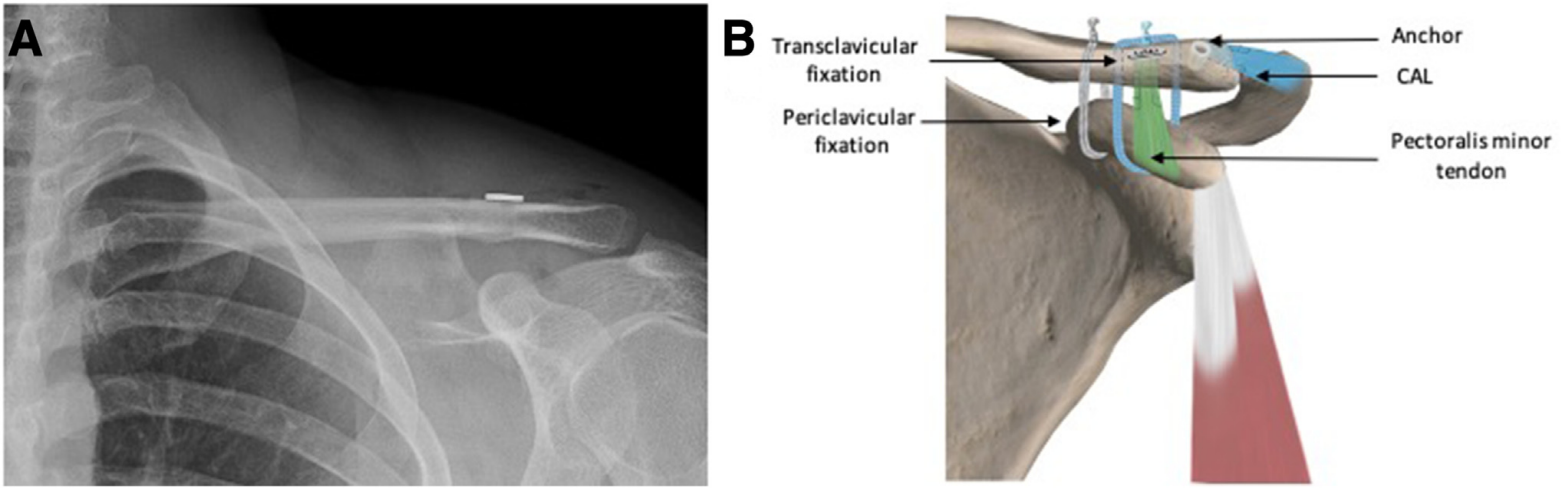
The Pm tendon sutures are secured in the superior surface of the clavicle over a button. The CAL sutures are passed under the deltoid fibers and fixed by an anchor in the posterosuperior cortex of the clavicle. (A) Postoperative radiograph of a left shoulder. (B) Postoperative illustration of a left shoulder. In green, the Pm tendon. In blue, the CAL fixed with an anchor. (CAL, coracoacromial ligament; Pm, pectoralis minor.)

**Fig 7:**
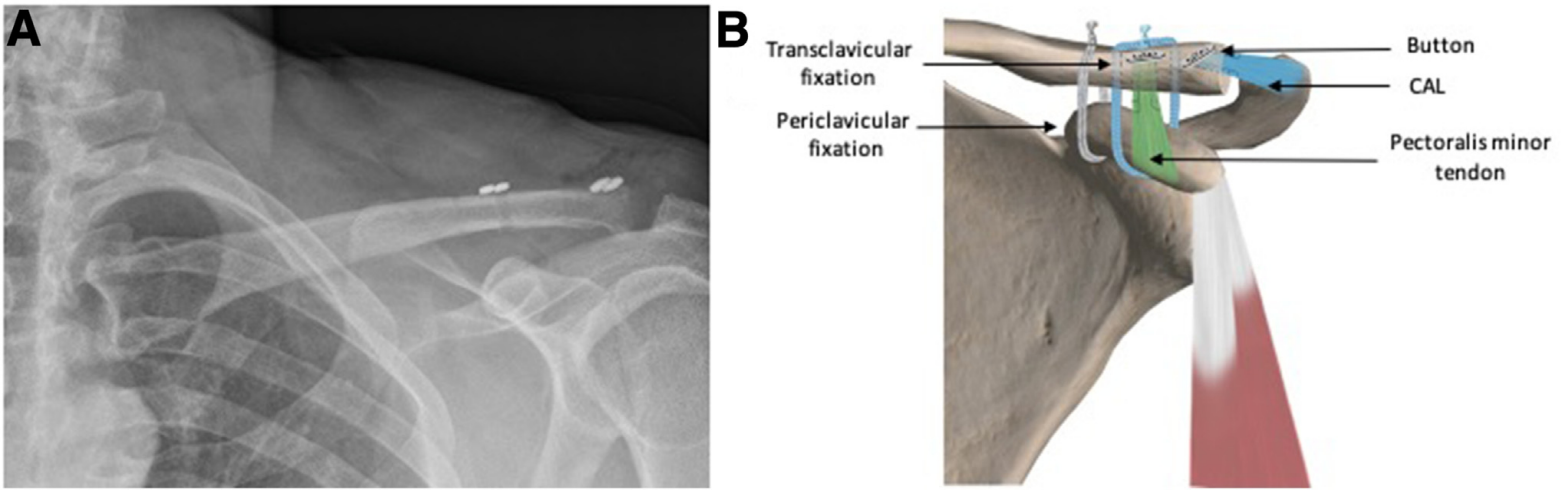
A 1-cm distal clavicle resection is performed in case of AC joint osteoarthritis or irreductible joints. In these situations, the CAL is fixed in the superior cortex by a button through a 2-mm tunnel. (A) Postoperative radiograph of a left shoulder. (B) Postoperative illustration of a left shoulder. In green, the Pm tendon sutures fixed with a button in the superior cortex of the clavicle. In blue, the CAL sutures fixed with a button. (AC, acromioclavicular; CAL, coracoacromial ligament; Pm, pectoralis minor.)

If it's necessary, a 1-cm distal clavicle resection and canal preparation is performed in order to receive the CAL transfer, which is fixed in the superior cortex by a button through a 2-mm tunnel.^7^ This procedure can be performed using the original incision or through a second mini-AC approach. Figures 6 and 7 illustrate the pre- and postoperative surgical results, respectively.

### Rehabilitation

Standar shoulder immobilization is used for 6 weeks. For the first 4 weeks, shoulder mobilization is restricted to passive assisted exercises, starting with active motion under supervision at the end of this period. Free active motion and strengthening exercises begin at week 6.

## Discussion

Chronic AC dislocations pose challenges as ligaments lose healing potential. The popular Weaver and Dunn technique, although widely used, yields suboptimal outcomes,^3^ leading to the development of more anatomical procedures. Carofino and Mazzocca’s anatomical CC reconstructive technique, which uses a semitendinosus graft, shows promising results.^5^ However, complications, including an 8.3% rate at the harvesting site and up to 28% local complications, limit the use of semitendinosus autografts.^6^^,^^8^ Allograft use, in contrast, is constrained by cost and availability.

Seeking alternatives to CAL transposition, Moinfar and Murthi^9^ conducted a biomechanical cadaveric study comparing the Pm tendon, CAL, and CC ligaments. They found that the Pm tendon has stronger vertical resistance and a wider musculotendinous junction, making it a potential local autograft for CC reconstruction. In addition, tenotomy of the Pm tendon is considered for scapular dyskinesis related to chronic AC dislocations.^10^

Although CAL transposition is commonly used as a vertical stabilizer of the AC joint, its potential role as a horizontal stabilizer has been less explored. Le Hanneur et al.^11^ introduced a “triple-bundle” reconstruction technique, detaching the CAL from the coracoid and securing it in the clavicle's medullary canal as a horizontal stabilizer. The feasibility and strength of this technique were demonstrated in cadaveric studies.^12^

The feasibility of the Pm tendon as a local autograft in association with the transfer of the CAL, for vertical and horizontal stabilization respectively, was first described in a cadaveric feasibility study by Cartaya et al.^13^ The Pm tendon graft has a stronger resistance to tensile force and a wider surface area in its tip compared with the CAL.^9^ Because it preserves the coracoid entesis, the Pm tendon graft also has the advantage of only needing one healing point. A combination of techniques, In contrast, has shown better multidirectional biomechanical stability.^14^ The use of the CAL as a horizontal stabilizer has been proven to be a strong configuration.^11^^,^^12^ Retaining CAL’s acromial insertion also has the benefit of preserving its role as a AC joint stabilizer.^7^ When the lateral clavicle resection is not needed, the fixation site of the CAL between the posterior one-third and the anterior two-thirds of the lateral clavicle was decided for 2 reasons. The first one was the less posterior translation on manual stress test compared with other points of fixation.^13^ The second reason was to create a potential biological augmentation to the posterosuperior AC joint complex.

Our procedure aims to preserve the lateral portion of the clavicle whenever possible. Grassbaugh et al.^15^ reported a significant difference in the revision rate of AC joint reconstructions, with a rate of 17% when the lateral clavicle was resected and 0% when it was preserved. Therefore, we propose limiting the resection of the lateral clavicle exclusively to cases of AC joint osteoarthritis or irreducible joints.

## Disclosures

All authors (M.C.M., F.T.R-T., J.V.Z.) declare that they have no known competing financial interests or personal relationships that could have appeared to influence the work reported in this paper.

## Supplementary Data

Video 1 A patient with Rockwood V chronic AC joint dislocation of the left shoulder is treated with the transfer of the Pm as a vertical stabilizer plus the transfer of the CAL as a horizontal stabilizer. (AC, acromioclavicular joint; CAL, coracoacromial ligament; Pm, pectoralis minor.)
